# Treatment of superficial infantile hemangiomas with timolol: Evaluation of short-term efficacy and safety in infants

**DOI:** 10.3892/etm.2013.1176

**Published:** 2013-06-21

**Authors:** LINJUN YU, SHENGMIAO LI, BAOLI SU, ZHENGJI LIU, JINGJING FANG, LIQI ZHU, MINYAN HUANG, WANGYONG SHAN, DAIQIANG SONG, BINBIN YE, CHUNFEN LUO

**Affiliations:** 1 Department of Pediatric Surgery, The Children’s Hospital, Zhejiang University School of Medicine, Hangzhou, Zhejiang 310006;; 2 Department of Pediatric Surgery, Taizhou Hospital, Wenzhou Medical College, Linhai, Zhejiang 317000, P.R. China

**Keywords:** timolol, infant, hemangioma, prospective study

## Abstract

Timolol has been demonstrated to be efficacious in the topical treatment of superficial infantile hemangiomas (IHs). We conducted a prospective study to evaluate the short-term efficacy and safety of timolol in the treatment of superficial IH in Chinese infants. From March to November 2012, 124 patients with superficial IHs were included in the prospective study. The patients were divided into two groups: treatment (101 patients, the timolol drops were administered on the surface of the lesions three times daily, and erythromycin ointment was applied around the lesions) and observation (23 patients, without treatment). The results were categorized into three grades: class 1 (ineffective), class 2 (controlled growth) and class 3 (promoted regression). Within one week of the initiation of timolol treatment, a number of the lesions became softer and lighter in color. Four months following the initiation of timolol treatment, the overall response was class 1 in eight patients (7.9%), class 2 in 36 patients (35.6%) and class 3 in 57 patients (56.4%). Complete tumor regression was observed in 12 patients. No adverse effects were recorded during the treatment period. Among the patients in the observation group, there were 15 class 1 patients (65.2%), seven class 2 patients (30.4%) and only one class 3 patient (4.3%). In conclusion, timolol is an effective and safe treatment for superficial IH. In addition, it may be used in the treatment of proliferative superficial IH, particularly in infants within 6 months of age.

## Introduction

Infantile hemangioma (IH) is one of the most common benign tumors of infancy, with occurrence rates of 8.7 to 12.7%, and up to 60% of the tumors presenting in the head and neck region (1). The tumor has a unique life cycle that is divided into three phases: proliferative, involuting and involuted. Although IHs usually regress spontaneously and only a limited number of cases require treatment, the prognostic factors have not yet been elucidated. Therefore, intervention may be necessary during the early proliferative phase to avoid complications, such as hemorrhage, ulceration, disfigurement and associated dysfunctions, depending on the location and size of the IH. In addition, early intervention may relieve the mental burden of the parents and avoid casting an unpredictable psychological shadow on the child. Guo and Ni (2) first revealed successful outcomes following the use of timolol solution in the treatment of a 4-month-old infant with superficial capillary hemangioma of the eyelid in 2010. Moreover, timolol has been demonstrated to be efficacious in the topical treatment of superficial IH in numerous countries, in addition to China. The present study evaluates the efficacy and safety of the use of topical 0.5% timolol maleate drops in the treatment of cutaneous superficial IH in Chinese infants.

## Patients and methods

### Patients

The study was approved by the ethics committee of

Taizhou hospital, Wenzhou Medical College (Linhai, China), and informed consent was obtained from the parents of all patients. Children treated with topical timolol drops for cutaneous superficial IH between March and November 2012 or those who underwent observation alone were included in this prospective study. The patients received routine follow-ups in the outpatient clinic of Taizhou hospital, Wenzhou Medical College within one week and following four months of treatment. The inclusion criteria were as follows: age ≤12 months; cutaneous superficial IH diagnosed according to the Waner and Suen 1999 classification criteria (3); no history of prior treatment; tumor thickness ≤3 mm and no evidence of short-term regression. Children with bronchial asthma, sinus bradycardia and second- or third-degree atrioventricular block were not excluded from the study.

The exclusion criteria comprised: age >12 months; prior treatment; tumor thickness >3 mm and evidence of tumor regression in the short-term.

### Methods

In the treatment group, the timolol drops were administered on the surface of the lesions three times daily, and erythromycin ointment was applied around the lesions to prevent the timolol from leaking. The parents were informed about the potential adverse effects, such as sleep changes, mental disorder, local itching and ulceration, and were told of any clinical signs and symptoms to look out for. The patients were photographed once a week, and any adverse effects, as well as changes in tumor color and size, were recorded. At the one-week and four-month follow-up examinations, the efficacy and safety of the timolol drops were evaluated.

The results were categorized into three classes compared with the baseline photographs. These were: class 1, ineffective, i.e. the lesion continued to grow; class 2, controlled growth, i.e. the lesion stopped growing and showed no significant change in size, color or texture; and class 3, promoted regression, i.e. the lesion became smaller, softer and lighter in color. The regression rate represented the percentage of cases with class 3 results, while the efficacy rate represented the percentage of cases with class 2 or class 3 results. The χ^2^ and Fisher’s exact tests were used to compare the response rates across the groups. P<0.05 was considered to indicate a statistically significant result.

## Results

**Figure 1.**
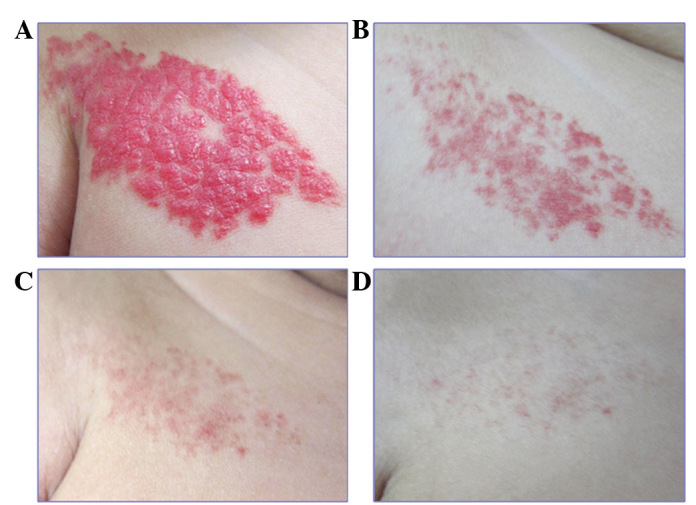
Topical timolol for the treatment of infantile hemangiomas. Hemangioma on the right chest wall of a 6-week-old female (A) prior to timolol treatment and after (B) one month, (C) three months and (D) four months of timolol treatment.

**Figure 2.**
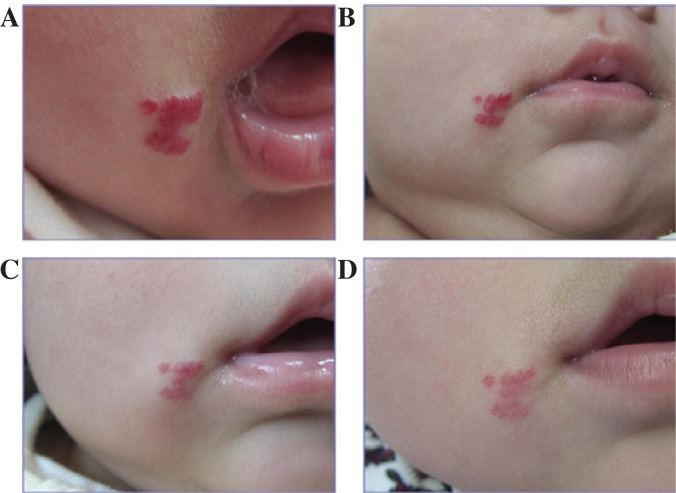
Topical timolol for the treatment of infantile hemangiomas. A 13-week-old female with a hemangioma on the right maxillofacial region (A) prior to timolol treatment and after (B) one month, (C) three months and (D) four months of timolol treatment.

A total of 124 patients with superficial IH from March to November, 2012, were included in this prospective study (Table I). Of these, 101 received treatment with topical timolol, and 23 underwent observation only. At one week subsequent to the initiation of timolol treatment, a number of tumors in the treatment group became softer and lighter in color, and at four months following the initiation of treatment (Figs. 1 and 2), the overall response was class 1 in eight patients (7.9%), class 2 in 36 patients (35.6%) and class 3 in 57 patients (56.4%; Table II).

Complete regression was observed in 12 patients, who stopped receiving the drug and showed no relapse during a 3–5-month follow-up period. There was no significant difference in efficacy rates between 1- to 6-month-old patients and 7- to 12-month-old patients (P>0.05); however, the regression rate in the 1- to 6-month-old patients was significantly higher than that in the 7- to 12-month-old patients (P<0.05). No adverse effects were reported. In the observation group, there were 15 class 1 patients (65.2%), seven class 2 patients (30.4%) and one class 3 patient (4.3%). The regression and efficacy rates of the treated group were significantly improved compared with those in the observation group (P<0.05).

## Discussion

Guo and Ni (2) were the first to reveal successful outcomes following the use of timolol solution in the treatment of a 4-month-old infant with superficial capillary hemangioma of the eyelid in 2010, and this success was subsequently reinforced by a series of other studies (4–7). However, to date, there have been relatively few studies on timolol treatment for IH, and the majority of these are case reports. Semkova and Kazandjieva (8) described the initial phase of a prospective study, which evaluated the efficacy and safety of topical 0.1% timolol gel for patients with IH. The patients were evaluated at 4-week intervals using the physician’s Global Assessment Score (GAS), and the mean result was an 85% improvement from baseline. Chambers *et al* (9) performed a retrospective, consecutive, nonrandomized, comparative single-masked cohort study, in which a good response (61.5%) was observed in the treatment group. By contrast, 0% of the observation group demonstrated a successful response. To date, the study by Chambers *et al* has been one of few to be undertaken in this field of research in the USA. Ye *et al* (10) investigated the efficacy and adverse effects of topical timolol maleate in the treatment of 12 periocular hemangiomas in a prospective study and observed that the control and regression rates were 83.3 and 50.0%, respectively. Following three months of treatment, 92.1% efficacy and 56.4% regression rates were observed. These rates were higher than those in the observation group. In the present study, erythromycin ointment was applied to prevent timolol leakage around the lesions and this may have increased the duration of drug action and improved the drug efficacy. The regression rate for the 1- to 6-month-old patients was significantly higher than that for the 7- to 12-month-olds; however, there was no significant difference in the efficacy rate. A further study with a large sample and a long follow-up period is required to confirm the efficacy of the treatment.

Timolol is a nonselective topical β-adrenergic antagonist that has been used in ophthalmology for >30 years. The predominant adverse effects include hypotension, hypoglycemia, bronchospasm and local pruritus. In the present study, the drug was topically applied to the skin of patients and, therefore, there was likely to be minimal absorption of the drug into the bloodstream. As expected, no systemic adverse reactions were observed during the treatment period of the study. Furthermore, no adverse effects have been reported in previous studies (2,7,8), with the exception of mild pruritus over the lesion following four weeks of treatment in a case where timolol application was used to treat an 18-month-old female with ulcerated hemangiomas associated with PHACE syndrome (5). In the experience of the authors, patients with bronchial asthma have not experienced attacks during treatment, and it is therefore proposed that timolol may be safely considered for use in the treatment of IH with bronchial asthma, sinus bradycardia and second- and third-degree atrioventricular block; however, there is a requirement for these patients to be observed closely during treatment. At present, there is a lack of relevant pharmacokinetic studies. Furthermore, the optimal drug dose and duration of treatment remain unclear.

Topical timolol treatment in superficial IH presents numerous advantages, and appears particularly useful as an effective, safe and relatively convenient treatment for this subtype of IH in patients up to 6 months of age.

## Figures and Tables

**Table I.** Patient characteristics.

Characteristic	Value
Age	
1–6 months (n)	88
7–12 months (n)	36
Gender	
Male (n)	47
Female (n)	77
Tumor size (mm)	5×5-60×85
Tumor thickness (mm)	0.5-3.0
Location	
Head and face (n)	65
Trunk (n)	27
Extremities (n)	32
History	
Bronchial asthma (n)	3
Total patients (n)	124

**Table II.** Efficacy of topical timolol in the treatment of superficial infantile hemangiomas.

Results	≤6 months old	>6 months old	Total
Class 1 (n)	5	3	8
Class 2 (n)	21	15	36
Class 3 (n)	46	11	57
Efficacy rate (%)	93.1	89.7	92.1
Regression rate (%)	63.9	37.9	56.4
